# Silence in physician clinical practice: a scoping review protocol

**DOI:** 10.1371/journal.pone.0307620

**Published:** 2026-03-17

**Authors:** Martina Ann Kelly, Stefanie Rivera, Caitlin McClurg, Catherine Sweeney, Stephen Mosca, Ellen McLeod, Deirdre Bennett, Megan Brown

**Affiliations:** 1 Department of Family Medicine, Cumming School of Medicine, University of Calgary, Calgary, Alberta, Canada; 2 Libraries and Cultural Resources, University of Calgary, Calgary, Alberta, Canada; 3 Medical Education Unit, School of Medicine, Brookfield Health Sciences Complex, University College Cork, Cork, Ireland; 4 Division of Palliative Care, University of British Columbia, Kelowna, Canada; 5 Division of Palliative Care, University of Calgary, Calgary, Alberta, Canada; 6 School of Medicine, Newcastle University, Newcastle, United Kingdom; Xiamen University - Malaysia Campus: Xiamen University - Malaysia, MALAYSIA

## Abstract

**Objective:**

The objective of this review is to map, describe and conceptualize how silence is discussed within literature on interactions between physicians and patients, in clinical settings.

**Methods:**

We will use the methodological framework of Arksey & O’Malley, adapted by Levac et al and Joanna Briggs Institute. Empirical studies including quantitative, qualitative, mixed methods, observational studies and reviews will be included. Commentaries, editorials, and grey literature will also be examined. The databases MEDLINE, Cumulative Index to Nursing and Allied Health Literature, PsycINFO, Scopus and Web of Science will be searched. A two-part study selection strategy will be applied. First, reviewers will follow inclusion and exclusion criteria based on ‘Population-Concept-Context’ framework to independently screen titles and abstracts. Next, full texts will be screened. Data will be extracted, collated, and charted to summarize methods, outcomes and key findings from the articles included. Findings will be reported following the Preferred Reporting Items for Systematic Reviews and Meta-analyses Extension for Scoping Reviews. (PRISMA-ScR).

**Expected results and implications:**

This scoping review will provide an extensive description of how physicians engage with silence in clinical settings. Findings will identify how silence is perceived in physician patient interactions, the roles it plays, what factors influence use of silence and guide development of educational initiatives on use of silence in clinical settings.

## Introduction

Effective use, and interpretation, of silence is a sophisticated and important communication skill.^1^ Proficient use of silence is associated with enhanced empathy, understanding, thoughtfulness, and self-awareness [1,2], important for good medical practice [3,4]. Despite this, research into silence in healthcare communication is lacking and the topic is poorly covered in communication skills curricula [5,6]. Having an in-depth understanding of physicians’ use, and experiences of, silence, could apprise communication skills training to enhance good physician-patient communication.

Silence is not merely the absence of verbal communication; it functions in dynamic relation to speech, forming a communicative continuum [7,8]. In clinical contexts, silence is typically defined as a pause in verbal audio signal, lasting longer than required for turn-taking (approximately 2 seconds) [9]. However, silence carries meaning beyond its technical definition. Its communicative significance is shaped by context, ambient sounds, and surrounding utterances. Silence can signal reflection, empathy, or tension, depending on these factors. Moreover, silence is rarely ‘empty’ – it is often accompanied by non-verbal cues such as eye contact, gestures, movement, posture, and paralinguistic communication, all of which contribute to its role as an active form of communication [10–12]. Culture also plays a role in silence. For example, in Asian contexts silence can be a sign of respect and is also acknowledged as being full of meaning. In contrast, in Western cultures, especially North America, silence can be perceived more negatively, as a sign of unfriendliness or not being worthy – with an expectation that speakers must ‘add’ something to the conversation [13].

Silence can play many roles in a consultation. In day-to-day language, silence, as expressed through pauses, is used to organize speech, such as turn-taking. For example, a physician may pause to invite a response, giving a participant a moment to gather their thoughts and think a bit longer about the subject matter. Silence can also afford emotional acknowledgement, for example following a moment of gravity. Silence can be non-productive, as in awkward silences, when the information being communicated is ambiguous or poorly understood. An awkward silence can also arise in the context of uncertainty, or distraction/ inattention. Non-productive silence can also be hostile, as in the withholding of information or judgement – a ‘conspiracy of silence’, or to prevent the sharing of anxiety. To date, several authors have attempted to characterize silence, using different terms such as compassionate silence [14,15], connectional silence [9,15], profound silence [16] and awkward silence [14].

Despite its significance, silence as a focus of research in healthcare is relatively limited, primarily originating from psychotherapy or palliative care [14,17]. A 2008 meta-ethnography of silence identified 18 studies, of which only 4 were empirical studies, the remainder consisting of opinion pieces, or commentaries [17]. This review identified that studies drew on literature from psychology, communication, and spiritual traditions. More recently, researchers in oncology and palliative care have audio-recorded consultations documenting the epidemiology of silence, coding for frequency of silence, duration, and several authors propose varying typologies of silence, often related to the duration and purpose of the pause [9,14,18,19]. However, the relationship between silence, nonverbal cues, and verbal communication, and how they influence each other, remains unclear. This scoping review seeks to address this research gap by examining how silence is described and conceptualized in the clinical literature. This protocol delineates the procedures for conducting the review, guided by good practice and protocols for scoping review development [20].

The primary objective of this review is to identify, analyze and synthesize how silence is engaged in interactions between physicians (including physicians in training) and patients, in the clinical setting. This information will, we anticipate, be useful to enhance physician-patient interactions through communication skills training for medical education.

Review questions:

1. How is silence conceptualized in the clinical literature involving physicians and patients?
2. What roles/ functions does silence play in physician-patient interactions?

## Methods

We chose to conduct a scoping review given the breadth of ways in which silence can be engaged. Scoping reviews are well suited to answer broad and exploratory research questions. They are used to explore new research areas, to clarify key concepts and identify research gaps by mapping the literary landscape, elucidating methodologies, core concepts, evidence types, and characteristics [21]. They frequently unveil a wider spectrum of evidence, serving as a foundation for systematic reviews and pinpointing knowledge voids [20,21]. This protocol has been reported using the Preferred Reporting Items for Systematic Reviews and Meta-analyses extension for systematic review protocols (PRISMA-P) [22] (S1 Appendix).

### Methodological framework

Our scoping review will follow the Arksey and O’Malley framework for scoping reviews [23], adapted by Levac et al. [24] and the Joanna Briggs Institute [25]. Components will include: identifying a research question; identifying relevant studies; study selection; charting data; collating, summarizing, and reporting results; and consultation. The findings of the review will be presented following the guidelines of the Preferred Reporting Items for Systematic Reviews and Meta-analyses extension for scoping reviews (PRISMA-ScR) [26].

### Stage 1: Identifying the research question

We used the Population Concept Context framework (PCC), recommended by the Joanna Briggs Institute for scoping reviews to develop our review question [25] (Table 1). We will focus our review on communication between physicians and patients, as these constitute a distinct form of interaction, central to the clinical encounter. This includes, for example, diagnostic reasoning, prognostic discussions, and treatment planning. While other healthcare professionals also play key roles in patient communication, including them would broaden the scope of the review, and may introduce heterogeneity in communication goals and relational dynamics, making it more difficult to derive insights that are specifically applicable to medical education. Further, the role of silence in physician-patient communication might be very different from that of other healthcare professionals due to variations in the professional hierarchies, responsibilities, and relationships with patients.

**Table 1:** Inclusion/ Exclusion criteria.

	Inclusion criteria	Exclusion criteria
Population	Physicians, ranging from general practitioners to specialists, resident physicians, and medical students.	Other healthcare professionals, such as Professional Nurses, Registered Nurses, Enrolled Nurses, Nurse Aides, Nursing Students, Dentists, Pharmacists, Nutritionists, EMTs, and Medical Laboratory Technologists. Additionally, Professional Therapists, Clinical Psychologists, Counseling Psychologists, as well as Professional Pastors and Spiritual Counselors
Concept	Silence as a communicative act, as verbally or non-verbally conveyed in conversation The role of silences in professional caregiving interactions with patients and clients. This involves analyzing how silence is used in clinical settings and communication skills curricula. These roles encompass silence in communication, verbal and non-verbal.	Silence in religious contexts Silence external to human interaction, e.g., nature Silence in relation to hearing impairment/ speech deficit Silence in relation to gene expression/therapy/drug treatments Electrocadaveric silence Silence and confidentiality/legal dimensions of practice Silence and noise in environment (e.g., Silencing beeping monitors) Silence and social media
Context	Clinical settings, such as hospitals, acute care settings, clinics, physician’s offices, emergency and urgent care centers, nursing homes, long-term care facilities, rehabilitation centers, mental health clinics, palliative care units, and home care environments Telemedicine.	Academic environments such classroom, simulations, lectures etc.

Our review question is ‘how is silence conceptualized in clinical literature involving physicians and patients?’ This question may be refined, or new ones added, as the authors gain increasing familiarity with the literature.

### Stage 2 Identifying relevant studies

#### Types of sources.

Empirical research on silence encompassing various study designs will be considered, including qualitative studies, observational studies, surveys and questionnaires, longitudinal studies, meta-analyses or evidence synthesis, and conversational analysis. We will also include commentaries, personal reflections and grey literature sources (conference proceedings, abstracts, thesis etc). Only English-language sources will be included. We deliberated this decision carefully but decided to exclude non-English-language studies, given the nuanced nature of silence in conversation, which often extends beyond the mere absence of speech and how engagement of silence varies across different cultural groups. There are no restrictions on publication dates.

A preliminary search was conducted on Google Scholar to gain an overview of existing literature and identify seed studies. Text word from titles, and abstracts of seed papers, along with the MeSH terms from MEDLINE were tailored to develop an initial search strategy for MEDLINE, S2 Appendix details our MEDLINE search strategy. Comprehensive searches will be carried out in the following databases: Scopus, Web of Science, CINAHL Plus with Full Text, APA PsychINFO, and MEDLINE. Reference lists of included studies will undergo screening to identify any additional relevant studies. The search strategy will be adapted for each database and further refined in consultation with a research librarian (CmC).

### Stage 3: Study selection

After completing the search, all located records will be uploaded to Covidence systemic review software (Veritas Health Innovation) and duplicates removed. A pilot test will be performed on a random subset of 50 titles/abstracts to refine the inclusion/exclusion criteria, if necessary, and to ensure consistent application of selection criteria among reviewers.

Screening will take place in 2 phases. All citations will be screened independently by 2 reviewers, based on title and abstract. Any discrepancies that arise during each stage of the selection process will be resolved by consulting a third reviewer. Next full texts will be imported into Covidence and reviewed by 2 independent reviewers. Reasons for exclusion will be documented. Findings of the search and the study inclusion process will be comprehensively reported in the final scoping review, following the reporting guidelines outlined in the Preferred Reporting Items for Systematic Reviews and Meta-analyses Extension for Scoping Reviews (PRISMA-ScR) [26].

### Stage 4: Charting the data

Before the extraction process, the team will collaborate to pilot five of the included studies to confirm accuracy, ensure mutual comprehension, and assess the suitability of the data extraction tool. A preliminary version of the data extraction tool is provided in Table 2. Modifications to the tool will be made as necessary and additional categories may be identified during data extraction. Any modifications will be carefully noted and disclosed as part of our audit trail. In instances where essential data for extraction is not readily available within the published paper, authors of the respective publications will be contacted for clarification.

**Table 2:** Preliminary Data Extraction Tool.

Categories	Questions
Publication Details	
Author(s)	Who are the authors of the publication?
Year of Publication	When was it published
Country of origin	Where was the study carried out?
**General Overview of Study**	
Objective and aims	What was the objective and aims of the study?
Methodology	Theoretical framework (if used)
	What methods (e.g., qualitative, quantitative, mixed methods) were used?
	How were patients and/or physicians engaged in the design process?
**Population**	Who were the participants, sample size?
**Concept**	What type of data concerning silence was included?
	Type of silence – duration, characteristics, e.g., in what manner is silence described, and characterized including the terms used, such as invitational, hostile, etc.?
	What, if any factors influence engaging with silence
**Context**	In what setting did the employment of silence take place?
	Cultural context/considerations – is culture mentioned, what role does it play in how silence is conceptualized?
**Findings/results**	What are the roles, purposes, or functions of silence?
	Any challenges/limitations reported?

### Stage 5: Collating, summarizing and reporting the results

Scoping reviews, differing from systematic reviews, generally do not evaluate the methodological quality or bias risk of the studies included, nor do they perform data synthesis like meta-analyses. Instead, they offer a descriptive overview of the studies encompassed [20]. Our approach involves both numerical and narrative summarization of various aspects of the included studies (Table 2). This analysis aims to map how silence is described in clinical interactions between patients and physicians. We anticipate that most studies will relate to Western clinical settings. Studies from non-Western contexts will be interpreted with explicit attention to their cultural settings. Silence will be treated as culturally mediated, and cross-study comparisons will be made only at a conceptual level to avoid imposing Western norms in communication.

Qualitative data will be analyzed using reflexive thematic analysis [27,28]. Two research team members will work independently to code the data inductively. Following this, the team will meet to define codes and compile a code-book. The team will meet regularly to discuss and refine coding, to ensure consistency across coders. Discrepancies will be resolved by consensus at a team meeting. Candidate themes will be documented at team meetings and refined throughout the analysis as the team works iteratively to identify patterns of shared meaning in the data extracted. Team reflexivity will draw on our experience as family physicians (MK, CS, DB), palliative care physicians (SM, EmL) and medical educationalists (MB) working across different health contexts and countries. We will draw on strategies outlined by Crabtree and Miller to aid our reflexivity [29]. We anticipate the need for regular team meetings to facilitate data charting and analysis and will maintain an audit trail to ensure that our final interpretations can be linked back to the data [24].

### Stage 6: Consultation

In line with Levac et al.‘s recommendation, we will integrate consultation as a component of our planned scoping review. [24] The outcomes of this review will be shared through presentations to palliative care physicians, ensuring a comprehensive exploration and understanding of silence’s role in clinical healthcare settings. This consultation phase will gather insights into our initial findings and their significance, explore potential applications and dissemination strategies, and identify areas requiring further research. Through this collaborative approach, we will facilitate discussions on implications and practical insights, e.g., to improve communication skills training. Engaging in conversations with physicians will offer valuable insights into the practical implications of the research, enriching our skill set by incorporating diverse perspectives into the research process.

## Limitations

Whilst we will do our best to identify all relevant literature, it may be that our search strategy may miss some studies. Additionally, our search is restricted to English language studies, this exclusion could potentially lead to missing relevant cultural and regional perspectives on silence in healthcare communication, which limits the generalizability of our findings.

This protocol is restricted to physicians and physicians in training. By limiting the scope to physicians, the findings are more likely to have clear implications for medical curricula, communication skills training, and clinical practice, which may not be generalizable to other healthcare professions. Additionally, our findings may not inform silence in interprofessional settings. This could be a focus for future research. We anticipate study heterogeneity, which may limit the type of analysis possible.

## Discussion

This protocol presents the methodological framework and approach we will employ to identify and map the existing evidence regarding the experiences of physicians with silence in clinical settings. By identifying, analyzing, and synthesizing existing literature on this topic, the review will offer insights into the various roles and functions of silence in physician-patient interactions. The review will help recognize the diverse roles that silence plays in clinical consultations. These roles encompass diverse aspects such as invitational silence, emotional acknowledgment, non-productive silence, and hostile silence, among others. Understanding these roles holds the potential to enhance the communication skills of healthcare professionals, enriching patient care experiences, improving their quality of life, and fostering a safe and comfortable environment within healthcare settings. Such insights can inform the development of tailored educational initiatives aimed at augmenting physicians’ proficiency in communication. Through dissemination via peer-reviewed presentations and publications, the findings of this scoping review will contribute to ongoing dialogues on optimizing physician-patient communication and refining healthcare delivery practices.

## Supporting information

S1 Appendix PRISMA-P Flow Chart. (DOCX)

S2 Appendix Search strategy terms, MEDLINE. (DOCX)
