# Supplementary material for: Silence in physician clinical practice: a scoping review protocol
Source: PLoS One. 2026 Mar 17;21(3):e0307620. doi: 10.1371/journal.pone.0307620 (PMC12994819; doi:10.1371/journal.pone.0307620)
Supplement: S1 Appendix — (DOCX) [file pone.0307620.s001.docx]

**S1: Appendix 1: PRISMA-P Flow Chart**

Studies from databases/registers **(n = 3233)**

Scopus (n = 1018)

Web of Science (n = 903)

PsycINFO (n = 487)

MEDLINE (n = 487)

CINAHL (n = 338)

References from other sources **(n = )**

Citation searching (n = )

Grey literature (n = )

**Identification**

Included studies ongoing **(n = 0)**

Studies awaiting classification **(n = 0)**

Studies included in review **(n = 101)**

Studies excluded **(n = 1677)**

Studies not retrieved **(n = 0)**

Studies assessed for eligibility **(n = 315)**

Studies sought for retrieval **(n = 315)**

Studies screened **(n = 1992)**

Studies excluded **(n = 214)**

Wrong setting (n = 2)

Not in English (n = 25)

Unable to find (n = 1)

Wrong population (n = 2)

Not an English Text (n = 1)

Wrong patient population (n = 1)

Focus on language studies (n = 2)

Not communicative silence (n = 131)

Not physicians (ie nurses, pscyhology etc) (n = 14)

Lacks insight into the role of silence in communication (n = 34)

editorial on study already included - duplicate material (n = 1)

References removed **(n = 1241)**

Duplicates identified manually (n = 61)

Duplicates identified by Covidence (n = 1180)

Marked as ineligible by automation tools (n = 0)

Other reasons (n = )

**Screening**

**Included**
