# Supplementary material for: Silence in physician clinical practice: a scoping review protocol
Source: PLoS One. 2026 Mar 17;21(3):e0307620. doi: 10.1371/journal.pone.0307620 (PMC12994819; doi:10.1371/journal.pone.0307620)
Supplement: S2 Appendix — (DOCX) [file pone.0307620.s002.docx]

**S2: Appendix: Search strategy terms, MEDLINE**

1 silence*.tw,kf.

2 discuss*.tw,kf.

3 support*.tw,kf.

4 relation*.tw,kf.

5 connect*.tw,kf.

6 exp Communication/

7 communicat*.tw,kf.

8 convers*.tw,kf.

9 interact*.tw,kf.

10 contemplat*.tw,kf.

11 acknowledg*.tw,kf.

12 paus*.tw,kf.

13 exp Mindfulness/

14 mindful*.tw,kf.

15 awkward*.tw,kf.

16 grie*.tw,kf.

17 peace*.tw,kf.

18 hope*.tw,kf.

19 exp "Attitude of Health Personnel"/

20 trust*.tw,kf.

21 interpersonal*.tw,kf.

22 exp Interpersonal Relations/

23 (bedside adj3 manner*).tw,kf.

24 manner*.tw,kf.

25 ?intent*.tw,kf.

26 ?comfort*.tw,kf.

27 unawar*.tw,kf.

28 talk*.tw,kf.

29 exp Voice/

30 voice*.tw,kf.

31 conspir*.tw,kf.

32 thematic.tw,kf.

33 textual.tw,kf.

34 unspoken.tw,kf.

35 tacit.tw,kf.

36 impli*.tw,kf.

37 quiet*.tw,kf.

38 exp Social Isolation/ or exp Loneliness/

39 solitude.tw,kf.

40 still*.tw,kf.

41 tranquil*.tw,kf.

42 2 or 3 or 4 or 5 or 6 or 7 or 8 or 9 or 10 or 11 or 12 or 13 or 14 or 15 or 16 or 17 or 18 or

19 or 20 or 21 or 22 or 23 or 24 or 25 or 26 or 27 or 28 or 29 or 30 or 31 or 32 or 33 or 34 or 35

or 36 or 37 or 38 or 39 or 40 or 41

43 exp Education, Medical/

44 exp "Internship and Residency"/

45 exp Faculty, Medical/

46 exp Physician-Patient Relations/

47 provider*.tw,kf.

48 clinician*.tw,kf.

49 trainee*.tw,kf.

50 residen*.tw,kf.

51 interns*.tw,kf.

52 preceptor*.tw,kf.

53 physician*.tw,kf.

54 doctor*.tw,kf.

55 (doctor adj4 relation*).tw,kf.

56 (physician adj4 relation*).tw,kf.

57 patient-physician.tw,kf.

58 (medic* adj3 educat*).tw,kf.

59 (medic* adj3 student*).tw,kf.

60 43 or 44 or 45 or 46 or 47 or 48 or 49 or 50 or 51 or 52 or 53 or 54 or 55 or 56 or 57 or

58 or 59 1

61 silent.tw,kf.

62 silenced.tw,kf.

63 silencing.tw,kf.

64 machine learning.tw,kf.

65 stigma*.tw,kf.

66 violen*.tw,kf.

67 abus*.tw,kf.

68 61 or 62 or 63 or 64 or 65 or 66 or 67

69 1 and 42 and 60

70 69 not 68
